# XOR‐Logic Phase Coding Programmable Metasurface for Low Power‐Consumption Systems

**DOI:** 10.1002/advs.202521960

**Published:** 2026-02-08

**Authors:** Ruichao Zhu, Sai Sui, Junyan Dai, Qunyan Zhou, Yuxiang Jia, Yajuan Han, Yuxi Li, Shaojie Wang, Qiang Cheng, Jiafu Wang, Tie Jun Cui

**Affiliations:** ^1^ Shaanxi Key Laboratory of Artificially‐Structured Functional Materials and Devices Air Force Engineering University Xi'an Shaanxi China; ^2^ State Key Laboratory of Millimeter Waves Southeast University Nanjing China; ^3^ SuZhou Laboratory SuZhou Jiangsu China

**Keywords:** low power consumption, phase coding, programmable metasurface, wireless communications, XOR logic

## Abstract

Programmability greatly enhances the degree of freedom to manipulate electromagnetic (EM) waves dynamically and lays crucial foundation for intelligent applications of metasurfaces. However, the traditional programmable metasurfaces need complicated biasing networks to control *m*×*n* digital meta‐atoms independently to fulfill the reprogrammable functions in real time, which also results in large power consumption to drive the metasurface. To alleviate this problem, we propose an XOR‐logic phase coding programmable metasurface to reduce the complexity of biasing network from *m*×*n* to *m+n*, which can reduce the power consumption significantly. The XOR‐logic phase coding is achieved by path symmetry of surface currents on a Pancharatnam‐Berry meta‐atom loaded with two PIN diodes. By controlling 2×*m*×*n* PIN diodes on the whole metasurface in row‐column manner, only *m+n* biasing lines are required to switch 0 and 1 states of all meta‐atoms independently. As the proof of concept, a prototype of the XOR‐logic phase coding programmable metasurface is designed and fabricated. Both simulation and measured results verify the reprogrammable functions of beam scanning and multi‐beam scattering. This work provides a new type programmable metasurface with simple architecture and low power consumption, which will find wide applications in intelligent systems such as next‐generation wireless communication, Internet of Things, and radar.

## Graphical abstract

By leveraging XOR logic, the biasing‐network complexity of 2D programmable metasurfaces is reduced from *m*×*n* to *m*+*n* through efficient row‐column control. This enables versatile functions including beam deflection and beamforming, facilitating applications in wireless communications and beyond.

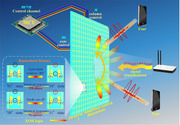

## 1 Introduction

Metasurfaces are 2D metamaterials, which are composed of the artificial structural units, exhibiting fascinating properties in manipulating electromagnetic (EM) waves. Metasurfaces have the high degree of freedom in modulating the EM‐wave amplitude, phase, polarization, and other properties [1, 2, 3]. In the past few decades, metasurfaces have made great progress as a means to control the EM waves and derived many intriguing physical phenomena. Therefore, some EM and optical devices, such as meta‐lens [4], invisible cloak [5], holographic imaging [6], can be implemented by metasurfaces. With the popularization of intelligence, the reconfigurable performance of functional metasurface has been widely concerned. Programmable metasurface breaks through the functional integration of the original space, and can quickly switch functions in the time dimension [7, 8].

By introducing materials or devices that can dynamically respond under external excitation into the metasurface, the control of equivalent EM response can be realized [9]. Common materials include graphene [10], vanadium dioxide [11], liquid crystals [12], etc. And these materials regulate the characterization of materials by changing excitation fields such as external electric field and temperature field, thus causing changes in electromagnetic response [13]. Moreover, the common devices include diode [14], micro electro mechanical system (MEMS) [15], etc. By changing the power supply excitation or external stress, the equivalent EM parameters of the unit can be changed to manipulate the EM response [16].

The concept of reconfigurable arrays was already evident in early studies. For example, mechanical [17] and electrical [18] tuning enabled control of microwave antenna arrays, laying the groundwork for the later informatization of metamaterials. In 2014, Giovampaola and Engheta proposed the concept of “digital metamaterials,” in which judiciously arranged spatial blends of “metamaterial bits” form elemental “metamaterial bytes” that deliver the targeted effective‐medium parameters [19]. Also in 2014, the concept of coding, digital, and programmable metamaterials were proposed to establish the bridge from the conventional metamaterials to information metamaterials, which provides convenience for digital control of EM waves [20]. At microwave frequencies, the PIN diode and varactor diode are generally loaded in the metasurface to change the equivalent circuit to control the equivalent EM response. Furthermore, with the control chip such as field programmable gate array (FPGA), the coding metasurface can realize digital representation and dynamic control [21, 22, 23]. Empowered by the concept of digital metamaterials, the coding metasurface delivers quasi‐electromagnetic functionality that is adequate for the majority of real‐world applications [24]. By digitally coding the metasurface, beam deflection [25, 26], scattering control [27, 28], focusing [29, 30], imaging [31, 32, 33] and other functions can all be digitally characterized. Moreover, the programmable metasurface can simulate the diffractive neural network to handle various tasks for wave sensing [34], including image classification [34, 35, 36], wireless communication [37], logical operation [38, 39], and so on. Furthermore, the integration of programmable metasurfaces with intelligent algorithms such as machine learning has forged a new paradigm of hardware‐algorithm co‐optimization [23, 40]. It is worth noting that most metasurfaces realize the precise control of the meta‐atoms through an extra biasing network with complex design. And the complexity of the meta‐atom's biasing network soars and even becomes prohibitive as array size, polarization patterns, and bits increase. Therefore, behind the exquisite function is the complex biasing network. However, more complex functions usually need 2D regulation, and the complex design of biasing network hinders the large‐scale arrays design of the metasurfaces. Owing to the requirement for independent control of 2D array, the number of feed ports required is proportional to the number of elements in the array. In general, the integrated ports of control chips such as FPGA are limited. Therefore, the more control chips need to be introduced to supplement these ports, thus causing heavier hardware consumption and more complicated biasing network for programmable metasurfaces. In previous studies, many researchers proposed various strategies to simplify the array design, including streamlined row‐column control and indirect addressing schemes. Li et al. realized row‐column cross‐addressing of a terahertz metasurface via liquid‐crystal tuning and demonstrated beam steering with the resulting array [41]. Sabri et al. achieved row‐column index addressing through a vertically layered voltage‐control design and demonstrated beam steering in the mid‐infrared range [42]. The schemes above are aimed chiefly at terahertz and higher frequencies. In the microwave band, the lattice period is comparatively large, thus both unit‐cell geometry and array‐level integration can be engineered in far greater detail. In the microwave regime, researchers have derived the theoretical foundations for cross‐addressing and designed the corresponding unit cells for both array antennas [43] and metasurfaces [44]. However, for the programmable metasurfaces at microwave frequencies, active devices such as PIN diodes and varactors, whose inherent unilateral conduction is widely exploited, render the cross‐addressing schemes reported in the aforementioned papers inapplicable without modification. Consequently, more refined unit‐cell engineering is urgently needed to counteract this asymmetry and thereby simplify the overall biasing network.

**FIGURE 1:**
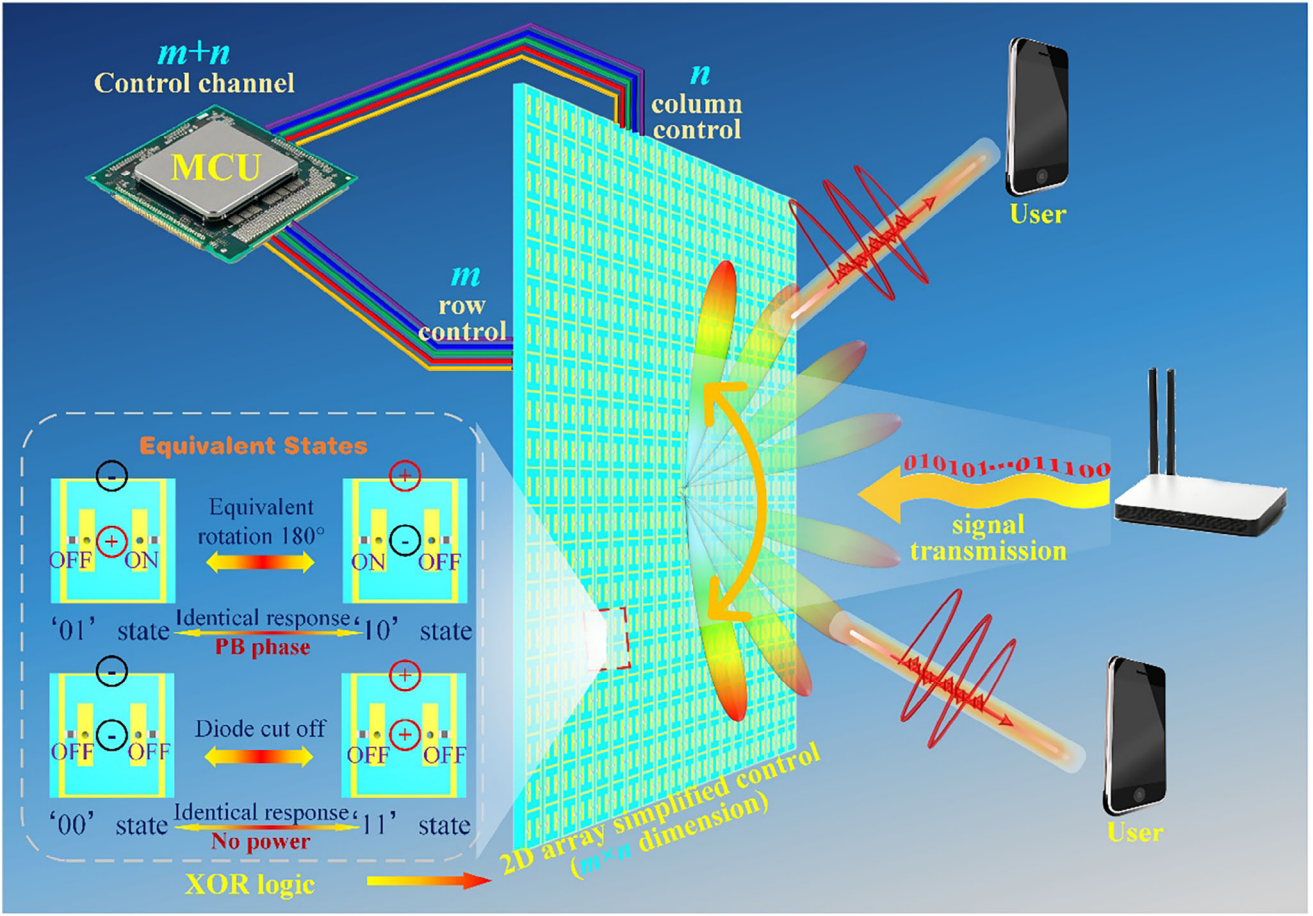
Schematic diagram of XOR logic simplified metasurface design.

In this work, we propose the XOR logic to simplify the biasing network design of 2D programmable metasurface (2D‐PM). Figure 1 illustrates the schematic diagram of this work, in which the XOR logic is applied in metasurface simplified design and applications. Leveraging the XOR logic control, the metasurface array can realize functions such as beam deflection and beamforming, thereby enabling applications in communications and other scenarios. The XOR logic meta‐atom includes two PIN diode to control the different EM response. The XOR logic of meta‐atoms is realized by customizing the path symmetry of surface current. The reason of this phenomenon is explained by Pancharatnam‐Berry (PB) phase theory. With this design, the meta‐atom obtains “0” phase response when the two diodes are in the same state (both ON or OFF), and “1” phase response when the two diodes are in different states (one ON and the other OFF). The XOR logic design transforms the complex 2D control into simple row and column control, which significantly reduces the number of feed ports. As verification, the 2D multi‐beam scattering control is designed through this 2D‐PM. The performance of multi‐beam is verified by simulation and measurement. Meanwhile, the 1‐bit phase coding can be assigned to each meta‐atom independently by XOR logic and the dimensionality of biasing network is reduced from *m*×*n* to *m*+*n*, which will significantly simplify the design and practical implementation of 2D‐PMs. The discussion of biasing simplification is supplemented in Note S1. In the field of communication, the XOR logic control method can improve the robustness of the feeding network and assist the metasurface to complete the blind communication. All the results are consistent well with theorical design, which fully demonstrates our design method. This work significantly simplifies the biasing network design of programmable metasurface and paves the way for large‐scale metasurface array design. Moreover, the simplified design method for digital control has wide application prospect in wireless communication and intelligent control.

## 2 XOR Logic Simplified Metasurface Design

### 2.1 Meta‐Atom Design

**FIGURE 2:**
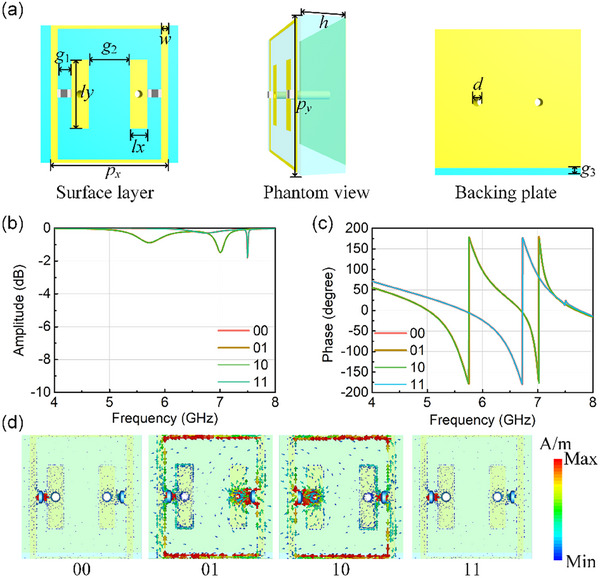
Meta‐atom design and analysis: (a) geometrical parameters and components of the meta‐atom; (b) the amplitude response at different states; (c) the phase response at different states; (d) the surface current distributions at different states.

Herein, the meta‐atom that can achieve XOR logic is designed as the control unit. The meta‐atom consists of three layers, that is, the surface function layer, the middle substrate layer, and the bottom metal reflector. The structure diagram and geometrical parameters of the XOR logic meta‐atom is shown in Figure 2a. The surface layer contains two rectangle‐shape patterns and a frame‐shape pattern with two PIN diodes loaded between the two structures. The gap between the two structures is *g*_1_ = 1mm. All the metal materials are the copper with the etching thickness is 0.017mm. The width of frame is *w* = 0.5mm. The horizontal length of frame is *p_x_* = 8.5mm and the vertical length of frame is *p_y_* = 10mm. The periodical size of meta‐atom also is *p_y_*, that is, the frame structure is connected in the vertical direction and can be used as a biasing line. The horizontal length and vertical length of rectangle‐shape pattern is *l_x_* = 1.25mm and *l_y_* = 5mm, respectively. The distance of two rectangle‐shape patterns is *g*_2_ = 3mm. The middle layer is F4B dielectric substrate with a dielectric constant 2.65(1+0.001j) and the thickness is *h* = 3mm. The bottom layer is metal reflector with a gap, which can be used as a biasing line while reflecting EM waves. The width of gap is *g*_3_ = 0.5mm. The surface layer and the back layer are connected by the metallized hole and the diameter is *d* = 0.6mm. The PIN diode is SMP1320‐079LF (from Skyworks), which can be equivalent by the equivalent circuit model composed of lumped elements at ON and OFF states [45, 46, 47]. According to the references, the equivalent parameters with *R* = 0.5Ω, *L* = 0.7nH is set to “ON” state and *L* = 0.5nH, *C* = 0.24pF is set to “OFF” state. We define the “ON/OFF” state as “1/0” and denote the bias state of the two diodes in the meta‐atom as a two‐digit binary character. When the frame‐shape pattern is connected to high or low level, the coded character on the left is set to 1 or 0. Similarly, when the rectangle‐shape is connected to high or low level, the coded character on the right is set to 1 or 0. Therefore, when the frame‐shape pattern is connected to high level, rectangle‐shape pattern is connected to low level, the left diode is switched on, and the right diode is cut off. In this case, we define the state as “10.” Similarly, the “00,” “01,” and “11” states can be obtained with different biasing state, respectively. The meta‐atom at different states is simulated by full‐wave simulation. The simulated amplitude and phase responses are shown in Figure 2b,c, in which the “00” and “11” states have the same EM responses and “01” and “10” have the same EM responses. Meanwhile, the different states have 180° phase difference at 5.8GHz. Therefore, the meta‐atom can implement logical calculations similar to XOR logic. In order to illustrate this phenomenon, the monitor at 5.8GHz is set to observe the surface current distributions which are shown in Figure 2d. In the “00” and “11” states, both the two diodes are turned off. Therefore, the two states have the same EM response. In the “01” and “10” states, the two diodes are in opposite states. In “01” state, the surface current forms a circulating current on the left side, whereas in “10” state, the surface current forms a circulating current on the right side. Therefore, from the surface current distributions, the path of surface current is symmetrical at the “01” and “10” states. In addition, the metal pattern is also centrosymmetric. Therefore, two different states of atoms can be viewed as rotated 180° around the center. For this specific unit designs, the control of two diodes has been simplified to a single control.

The phase response caused by unit rotation can be analyzed by Pancharatnam–Berry (PB) phase theory [48, 49]. Mathematically, the geometric phase is analyzed by Jones matrix. The more detailed analysis of the Jones matrix is presented in the Note S2. The Jones matrix of the reflected wave generated by the incident wave irradiating on the metasurface along the ‐*z* direction can be written as Equation (1):

(1) $$\left(\begin{array}{cc} R_{RL} & R_{RR}\\ R_{LL} & R_{LR} \end{array}\right)=\left(\begin{array}{cc} R_{xx}+R_{yy}+iR_{xy}-iR_{yx} & \left(R_{xx}-R_{yy}-iR_{xy}-iR_{yx}\right)\cdot e^{2\theta i}\\ \left(R_{xx}-R_{yy}+iR_{xy}+iR_{yx}\right)\cdot e^{-2\theta i} & R_{xx}+R_{yy}-iR_{xy}+iR_{yx} \end{array}\right)/2$$

in which the *R_LL_* and *R_RL_* are the reflection coefficients of the co‐polarization and cross‐polarization under the incidence of left‐handed circularly polarized (LCP) waves. Similarly, *R_RR_* and *R_LR_* represent the co‐polarization and cross‐polarization under the incidence of right‐handed circularly polarized (RCP) waves. From Equation (1), it can be concluded that the co‐polarized reflected wave generated by the incident of LCP wave carries an additional geometric phase of −2*θ*, and the co‐polarized reflected wave generated by the incident of RCP wave carries an additional geometric phase of 2*θ*.

Especially, the rotation angle of “01” state relative to “10” state is 180°. As inferred from Equation (1), the LCP component will obtain −360° reflected phase response and the RCP component will obtain 360° reflected phase response. Therefore, the rotation of 180° is equivalent to having no effect on the state of LCP and RCP waves. Moreover, any linearly polarized (LP) wave with the polarization direction *φ* can be decomposed into the superposition of two orthogonal circularly polarized (CP) components with equal amplitude [50, 51]. The superposition of CP can be expressed Jones vector as shown in Equation (2):

(2) $$|LP_{\varphi }\rangle =\left[\begin{array}{c} \cos\varphi \\ \sin\varphi \end{array}\right]=\frac{\sqrt{2}}{2}\left\{\frac{1}{\sqrt{2}}\left[\begin{array}{c} 1\\ -i \end{array}\right]e^{i\varphi }+\frac{1}{\sqrt{2}}\left[\begin{array}{c} 1\\ i \end{array}\right]e^{i-\varphi }\right\}=\frac{\sqrt{2}}{2}\left(e^{i\varphi }|L\rangle +e^{i-\varphi }|R\rangle \right)$$

where |*LP*φ〉 is the LP wave, |**R**〉 and |**L**〉 are the RCP wave and LCP wave, respectively. Owing to the fact that RCP and LCP components are equivalent to no phase change, the phase of the superposition LP wave has not changed either. Therefore, the “01” and “10” states have the same phase response. Based on this, the meta‐atom implements XOR logic.

### 2.2 Scattering Control

**FIGURE 3:**
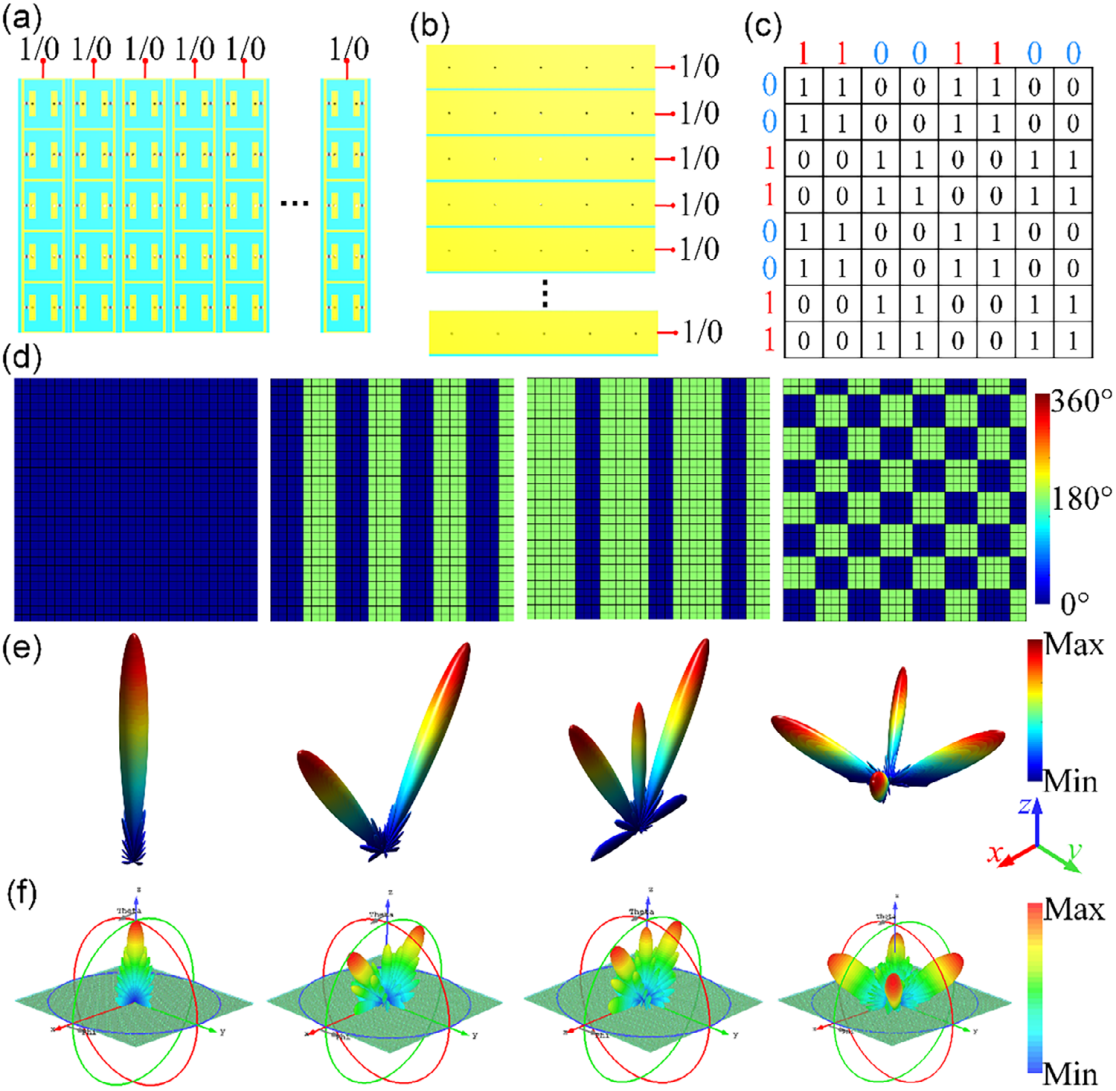
The biasing network design and scattering mode design: (a) The surface layer biasing mode is connected to control column's coding in 2D array; (b) The bottom layer biasing mode is connected to control row's coding in 2D array (c) The chessboard arrangement as example that is calculated by XOR logical operations; (d) The phase profiles for 1‐, 2‐, 3‐, and 4‐beam scattering mode; (e) The theorical calculation of 3D far‐field scattering patterns under the phase profiles; (f) The simulated 3D far‐field scattering patterns under the phase profiles.

Based on the above design, the working states of meta‐atom can be customized by changing the biasing voltage. The meta‐atoms are arranged periodically to form a metasurface array, in which the frame‐shape can achieve longitudinal feeding and the rectangle‐shape pattern connected to backplane can achieve horizontal feeding. The schematic diagram of longitudinal and horizontal feeding mode is shown in Figure 3a,b. According to the previous analysis, the designed meta‐atom has XOR logic. Therefore, the 2D array can be customized by changing the feeding mode of the rows and columns. As shown in Figure 3c, the chessboard array is set as an example to illustrate the XOR logic phase distributions, where the coding sequence of each point is calculated by XOR logic of the corresponding coding of rows and columns. Only the ports corresponding to rows and columns need to be directly connected to the control module, and the voltage of the output port can be controlled by the control chip to realize the regulation of the 2D array. The control module can choose FPGA, arduino, programmable voltage source and others to change the voltage distributions. The metausrface consists of 30 × 30 meta‐atoms arrays, which means that only 60 control ports are needed to realize 2D control. Compared to the original 900 control ports, the number of ports is reduced by about 96%.

With this control manner, the 2D coding array can be customized to modulate the scattering mode. The far‐field scattering pattern corresponding to different coding modes can be calculated according to Equation (3) [20, 52]:

(3) $$\begin{array}{cc} F\left(\theta ,\varphi \right) & =f_{m,n}\left(\theta ,\varphi \right)\sum_{m=1}^{M}\sum_{n=1}^{N}\exp\{j[\varphi_{m,n}+k_{0}D_{x}\left(m-1/2\right)\\ & \;\times \left(\sin\theta \cos\varphi -\sin\theta_{i}\cos\varphi_{i}\right)+k_{0}D_{y}\left(n-1/2\right)\\ & \;\times \left(\sin\theta \sin\varphi -\sin\theta_{i}\sin\varphi_{i}\right)]\} \end{array}$$

where *θ* and *φ* are elevation angle and azimuth angle, respectively. And *f_m_*_,_ *_n_* (*θ*,*φ*) is the pattern function which is a constant, *k_0_* is the vacuum wave vector, *M* and *N* are the dimensions of the array. *Φ_m,n_* is the phase of unit at position (*m,n*), *D_x_* is the horizontal length of the unit and *D_y_* is the vertical length of the unit. The phase coding mode of the array can be obtained by coding the rows and columns through the ports of the control board respectively and performing XOR operation. Furthermore, the far‐field scattering pattern can be calculated by Equation (3).

Here, we design different coding modes to realize multi‐beam respectively. Specifically, 1‐, 2‐, 3‐, and 4‐beam scattering modes are designed and verified, respectively. The more multi‐beam cases are supplemented in the Note S3. Additionally, we also supplemented the discussion here with the limitations of matrix decomposition and distributed control. And the application scenario of multi‐beam switching is supplemented in Note S4. Figure 3d illustrates the phase profiles of multi‐beam control. The metasurface is simulated under the four different phase profiles as shown in Figure 3d. All the boundary conditions are set to “open (add space).” The *x*‐polarized plane waves are incident perpendicularly to the metasurface. The far‐field monitor is set at 5.8GHz to observe the scattering mode. Figure 3e illustrates the 3D far‐field scattering patterns, which exhits the 1‐, 2‐, 3‐, and 4‐beam scattering pattern under the four different phase profiles. As shown, the metasurface system is capable of controlling multi‐beams. However, full‐aperture operation requires that the row‐column addressing satisfy the XOR‐logic condition. Although simultaneously arbitrary control of every individual pixel is not possible, because the per‐pixel state is constrained to XOR of its row and column codes, the proposed design can still approximate a variety of functional wavefronts.

## 3 Experiment and Test

**FIGURE 4:**
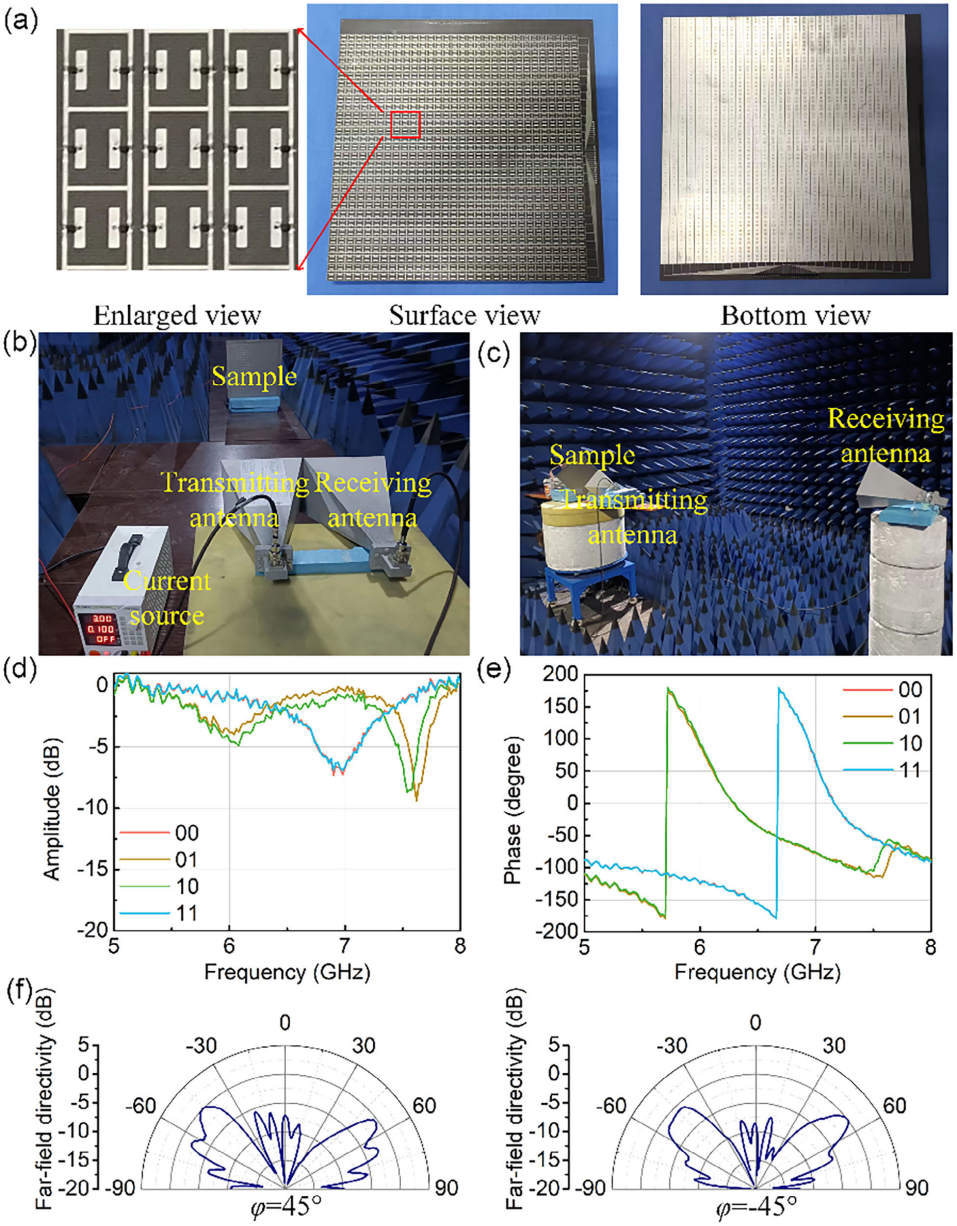
Fabrication and measurement: (a) the photograph of metasurface; (b) reflection coefficient measurement environment; (c) Far‐field measurement environment; (d) reflected amplitude responses in different states; (e) reflected phase responses in different states; (f) the far‐field directivity patterns measured by tilting diagonally at *φ* = ±45°.

Herein, the metasurface prototype is fabricated by commercial Printed Circuit Board (PCB) technology and the photograph is exhibited in Figure 4a. The biasing line network of engineering drawings are supplemented in Note S5, in which the surface pattern and reflective backboard are also the feeding port. The biasing network is designed as described above, that is, cross control is carried out through rows and columns respectively. The size of metasurface is 320 × 320mm including the extra biasing area. Two PIN diode (SMP1320‐079LF, Skyworks) are welded into each meta‐atom with SC79 packaging technology. The analysis of power consumptions isi presented in Note S6. The typical on/off value for this diode is 0.4 µs. Considering factors such as the settling time of the driving circuit and the influence of package parasitic parameters (the datasheet lists the package inductance *Ls*, e.g., 0.7 nH for SC‐79), the total switching stabilization time of the SMP1320 diode in an actual circuit is conservatively estimated to be in the range of 1–2 µs. The reflection coefficient measurement is shown in Figure 4b, in which the metasurface sample is measured in microwave anechoic chamber with a vector network analyzer (Agilent E8363B) and two horn antennas. The current source is connected to the metasurface to change the biasing states. In order to reduce the EM losses, the current is set to 100 mA (100 mA for 90 meta‐atoms, approximately, and 1.1 mA per unit cell) to reduce the internal resistance. The measured amplitude responses at different states are shown in Figure 4d, and the measured phase responses at different states are shown in Figure 4e. From the measured reflection coefficients, the EM responses of 00 and 11, as well as 01 and 10, are basically the same. A slight mismatch between 01 and 10 is caused by the non‐central symmetry of the biasing region. According to the measured reflection coefficient, the working frequency is around 6.1GHz with 180° phase difference at different states, which is only 0.3GHz offset from the simulation design. The slight discrepancy between measured and simulated operating frequencies most likely arises from fabrication tolerances, biasing‐line parasitics, and so on. A detailed discussion of these factors is provided in Note S7. Futhermore, the ability of scattering beam control is measured by far‐field measurement as shown in Figure 4c. The metasurface placed on rotating platform with transmitting antenna, anthor horn antenna as receiving antenna is set to record the scattering beams. Here, the beam control capability of the metasurface is verified only in the case of 4‐beam scattering, that is, the metasurface is intersected in a checkerboard mode. The metasurface and antenna were tilted to ±45° respectively to test the far‐filed directivity patterns under different azimuths. The measured far‐filed patterns are shown in Figure 4f, where the two beams are detected in *φ* = 45° and *φ* = −45°, respectively. The far‐field measurement resuls illustrated that the metasurface realized the 4‐beam scattering mode.

**FIGURE 5:**
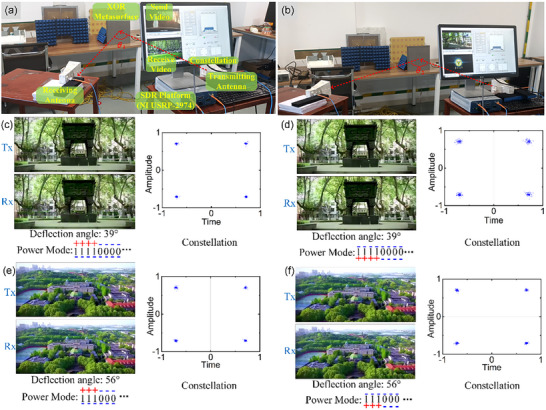
Wireless communication experiments: (a) measurement environment at reflection angle *θ*_1_ = 39°; (b) measurement environment at reflection angle *θ*_1_ = 56°; (c) The video is transmitted in the case of coding “111000…” in the forward conduction and the corresponding QPSK constellation; (d) The video is transmitted in the case of coding “111000…” in the reverse conduction and the corresponding QPSK constellation; (e) The video is transmitted in the case of coding “11110000…” in the forward conduction and the corresponding QPSK constellation; (f) The video is transmitted in the case of coding “11110000…” in the reverse conduction and the corresponding QPSK constellation.

To further illustrate the powerful capabilities of XOR‐logic phase coding programmable metasurface, the wireless communication experiments are conducted using a software‐defined radio (SDR) platform (NI USRP‐2974). The SDR platform can verify the RF transmission performance with a lower cost. The wireless communication function of metasurface is implemented through data transmission via bitstreams. A demonstration video is converted into bitstreams and transmitted by the wireless communication system through quadrature phase‐shift keying (QPSK) modulation. The test scenario of the communication experiment is shown in Figure 5a,b, in which the metasurface is placed on the test platform and two horn antennas are set as transmitter and receiver. The video is converted into bitstreams and transmitted experimentally through horn antennas. Two different coding cycles were tested separately, corresponding to different angles as shown in Figure 5a,b. According to the far‐field scattering formula (1), the theoretical scattering angles at different coding cycles are 39° and 56°, respectively. Due to the characteristic of XOR logic of the designed structure, the metasurface can maintain the same coding mode only by inversely connecting the positive and negative electrodes. Figure 5c–f illustrates the demonstration of video transmission, in which the screenshots verify the reliability of the transmitted content and the constellation verify the modulation of signal. The power mode of $\begin{array}{c} +\\ - \end{array}$ means that the positive electrode is connected vertically above and the negative electrode is connected vertically below. Oppositely, the power mode of $\begin{array}{c} -\;+ \end{array}$ means that the negative electrode is connected vertically above and the positive electrode is connected vertically below. Meanwhile, in both cases, the phase response of the unit is “1” coding state. Figure 5c,d shows the case where the coding period is 40 mm, and the “1” coding unit is implemented through two power supply modes. The signal transmission details at 40mm coding period in both power supply modes are demonstrated in Video S1. Analogously, Figure 5e,f show the case where the coding period is 30 mm in different power modes. The signal transmission details at 30mm coding period in both power supply modes are shown in Video S2. Furthermore, the comparison of signal communication between metasurface working and non‐working is given in Video S3, which can demonstrate the communication blind compensation function of the XOR metasurface. According to the above test results, the XOR metasurface can realize the communication function under different power supply modes, which verifies the power supply robustness of the metasurface in communication applications.

## 4 Conclusions

We propose the XOR logic to simplify the biasing network design of 2D programmable metasurface. Two symmetrical PIN diodes are embedded in the centrosymmetric meta‐atom to achieve the XOR logic and the phenomenon can be explained by PB phase theory. The XOR logic design transforms the complex 2D control into simple row and column control, which significantly reduces the number of feed ports of control devices. The XOR logic design is verified by the function of multi‐beam scattering control. The effectiveness of the design is demonstrated by theoretical design, simulation calculation, and experimental verification. Most importantly, this work paves a new way for simplifying programmable metasurface design, which can be further extended in other application scenarios. Our work presents a control architecture. In future research, higher‐speed devices such as GaAs FET switches (with switching times on the nanosecond scale), MEMS switches (hundreds of nanoseconds), or ferroelectric material‐based capacitors (e.g., BST, nanosecond scale) could be employed, which would further enhance the system's speed.

## Conflicts of Interest

The authors declare no conflicts of interest.

## Supporting information




**Supporting File 1**: advs74287‐sup‐0001‐SuppMat.docx.

**Supporting File 2**: advs74287‐sup‐0002‐Video1.mp4.

**Supporting File 3**: advs74287‐sup‐0003‐Video2.mp4.

**Supporting File 4**: advs74287‐sup‐0004‐Video3.mp4.

## Data Availability

The data that support the findings of this study are available from the corresponding author upon reasonable request.
