# Supplementary material for: XOR‐Logic Phase Coding Programmable Metasurface for Low Power‐Consumption Systems
Source: Adv Sci (Weinh). 2026 Feb 8;13(22):e21960. doi: 10.1002/advs.202521960 (PMC13088299; doi:10.1002/advs.202521960)
Supplement: Supplementary file 1 — Supporting File 1: advs74287‐sup‐0001‐SuppMat.docx. [file ADVS-13-e21960-s001.docx]

Supplementary information for

**XOR-logic phase coding programmable metasurface for low power-consumption systems**

*Ruichao Zhu^1^, Sai Sui^1^*, Junyan Dai^2^, Qunyan Zhou^2^, Yuxiang Jia^1,3^, Yajuan Han^1,3^, Yuxi Li^1^, Shaojie Wang^1^, Qiang Cheng^2^, Jiafu Wang^1,3^*, Tiejun Cui^2,3^**

1 *Shaanxi Key Laboratory of Artificially-Structured Functional Materials and Devices, Air Force Engineering University, Xi'an, Shaanxi 710051, China*

2 *Institute of Electromagnetic Space, Southeast University, Nanjing, 210096, China*

3 *SuZhou Laboratory, SuZhou, Jiangsu 215000, China*

Corresponding author. E-mail: [suisai_mail@foxmail.com](mailto:suisai_mail@foxmail.com); [wangjiafu1981@126.com](mailto:wangjiafu1981@126.com；); [tjcui@seu.edu.cn](mailto:tjcui@seu.edu.cn)

The supplementary file includes:

**Note 1. Biasing simplification**

**Note 2. Jones matrix analysis**

**Note 3. Multi-beam control**

**Note 4. Metasurface empowered multi-target communication**

**Note 5. The biasing line network of engineering drawings**

**Note 6. Analysis of power consumptions**

**Supplementary Note 1. Biasing simplification**

This work provides the biasing network simplified design method, and the schematic diagram is shown in Figure S1. Owing to the requirement for independent control of two-dimensional array, the number of feed ports required is proportional to the number of elements in the array. Assuming that the two-dimensional metasurface array contains *m* rows and *n* columns, the metasurface units requiring single point control contain *m*×*n*. Therefore, the unit control circuit contained in the conventional two-dimensional reconfigurable metasurface is two-dimensional, which leads to the complexity of circuit design. However, the integrated ports of control chips such as FPGA are limited. Therefore, the more control chips need to be introduced to supplement these ports, thus causing higher hardware cost and more complex control circuit for reconfigurable metasurfaces.

Herein, the XOR logic simplified metasurface can control the two-dimensional reconfigurable array only by transverse and longitudinal cross-feeding. In this case, the required number of feeding ports is the number of rows plus the number of columns, that is, *m*+*n*. By designing the XOR logic metasurface feeding network, the design realizes the simplification of two-dimensional control network to one-dimensional control network, which lays a foundation for the further expansion of intelligent control system.


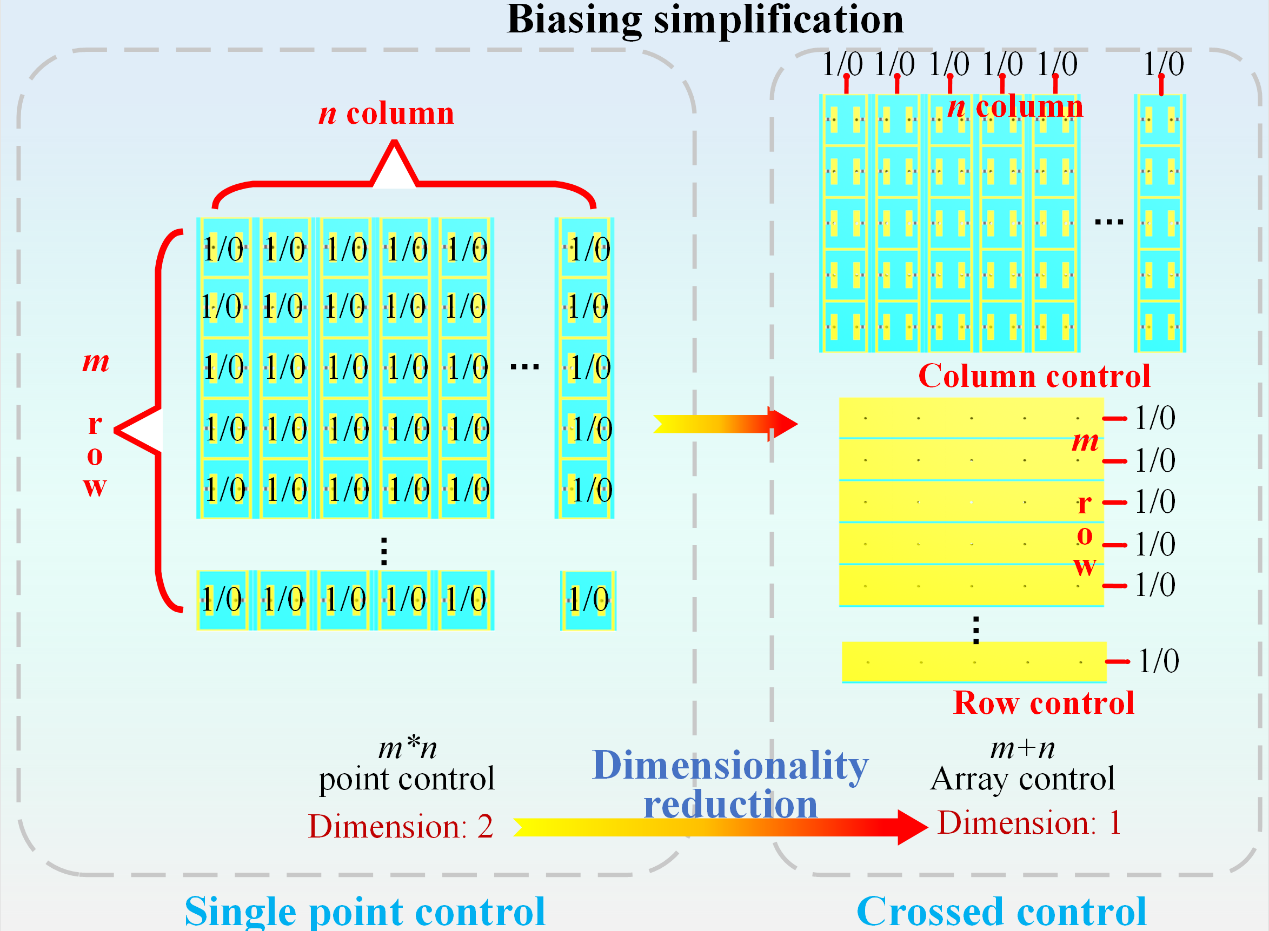


Figure S1 The schematic diagram of biasing simplification

**Supplementary Note 2. Jones matrix analysis**

When linearly polarized (LP) waves are irradiated on the metasurface along the -*z* direction, the relationship between the reflected electric field and the incident electric field can be described by Jones matrix *Rθ LP* as follows [1,2]:

$\left(\begin{matrix} E_{x}^{r} \\ E_{y}^{r} \end{matrix}\right)=R_{LP}^{\theta}\cdot\left(\begin{matrix} E_{x}^{i} \\ E_{y}^{i} \end{matrix}\right)=\left(\begin{matrix} R_{xx} & R_{xy} \\ R_{yx} & R_{yy} \end{matrix}\right)\cdot\left(\begin{matrix} E_{x}^{i} \\ E_{y}^{i} \end{matrix}\right)$ (S1)

in which *Et x* and *Et y* represent the reflected electric field components of *x* polarization and *y* polarization respectively, *Ei x* and *Ei y* represent the incident electric field components of *x* polarization and *y* polarization respectively, *R_xx_* and *R_yx_* represent the co-polarization and cross-polarization transmission coefficients of *x* polarization wave, while *R_yy_* and *R_xy_* represent the co-polarization and cross-polarization reflection coefficients of *y* polarization wave. When the structure rotates around the *z* axis by *θ*, the Jones matrix will be transformed into:

$R_{LP}^{\theta}=M^{-1}\cdot R_{LP}^{0}\cdot M=\left(\begin{matrix} \cos\theta& -\sin\theta\\ \sin\theta& \cos\theta\end{matrix}\right)^{-1}\cdot\left(\begin{matrix} R_{xx} & R_{xy} \\ R_{yx} & R_{yy} \end{matrix}\right)\cdot\left(\begin{matrix} \cos\theta& -\sin\theta\\ \sin\theta& \cos\theta\end{matrix}\right)$ (S2)

where *M* is the rotation matrix. The reflection electric field of the structure under the irradiation of circularly polarized (CP) wave can be expressed by the incident electric field and Jones matrix *Rθ CP* as follows:

$\left(\begin{matrix} E_{L}^{r} \\ E_{R}^{r} \end{matrix}\right)=R_{CP}^{\theta}\cdot\left(\begin{matrix} E_{L}^{i} \\ E_{R}^{i} \end{matrix}\right)=\left(\begin{matrix} R_{LL} & R_{LR} \\ R_{RL} & R_{RR} \end{matrix}\right)\cdot\left(\begin{matrix} E_{L}^{i} \\ E_{R}^{i} \end{matrix}\right)$ (S3)

in which *Er L* and *Er R* represent the reflected electric field components of left-handed circular polarization (LCP) and right-handed circular polarization (RCP) respectively, *Ei L* and *Ei R* represent the incident electric field components of LCP and RCP respectively, *T_LL_* and *T_RL_* represent the co-polarized and cross-polarized reflection coefficients of LCP, while *R_RR_* and *R_LR_* represent the co-polarized and cross-polarized reflection coefficients of RCP [3]. Therefore, the Jones matrix of the reflected wave generated by the incident wave irradiating on the metasurface along the -*z* direction can be written as equation (S4):

$\left(\begin{matrix} R_{RL} & R_{RR} \\ R_{LL} & R_{LR} \end{matrix}\right)=\left(\begin{matrix} R_{xx}+R_{yy}+iR_{xy}-iR_{yx} & (R_{xx}-R_{yy}-iR_{xy}-iR_{yx})\cdot e^{2\theta i} \\ (R_{xx}-R_{yy}+iR_{xy}+iR_{yx})\cdot e^{-2\theta i} & R_{xx}+R_{yy}-iR_{xy}+iR_{yx} \end{matrix}\right)/2$ (S4)

in which *R_LL_* and *R_RL_* represent the co-polarization and cross-polarization reflection coefficients under the incident of LCP and RCP waves. *R_xx_* and *R_yx_* represent the co-polarization and cross-polarization transmission coefficients of *x* polarization wave, while *R_yy_* and *R_xy_* represent the co-polarization and cross-polarization reflection coefficients of *y* polarization wave. Therefore, the same polarized reflection wave produced by the LCP wave incident carries -2*θ* additional geometric phase, and the same polarized reflection wave produced by the RCP wave incident carries 2*θ* additional geometric phase, but the cross-polarized reflection wave produced by the two circularly polarized wave incident does not have any additional geometric phase. When the rotation angle is 180 degrees, the phase variation of the LCP wave is -2×180°, and the phase variation of RCP wave is 2×180°. That is, phase variation of the LCP wave is -360° and RCP wave is 360°, which be equivalent to 0° and the resultant linearly polarized wave also causes a phase of 0°. Based on this PB phase theory, we realize the design of XOR logic.

**Supplementary Note 3. Multi-beam control**

The more multi-beam cases is shown in Figure S2. The phase profiles are searched randomly to generated the different scattering beams.


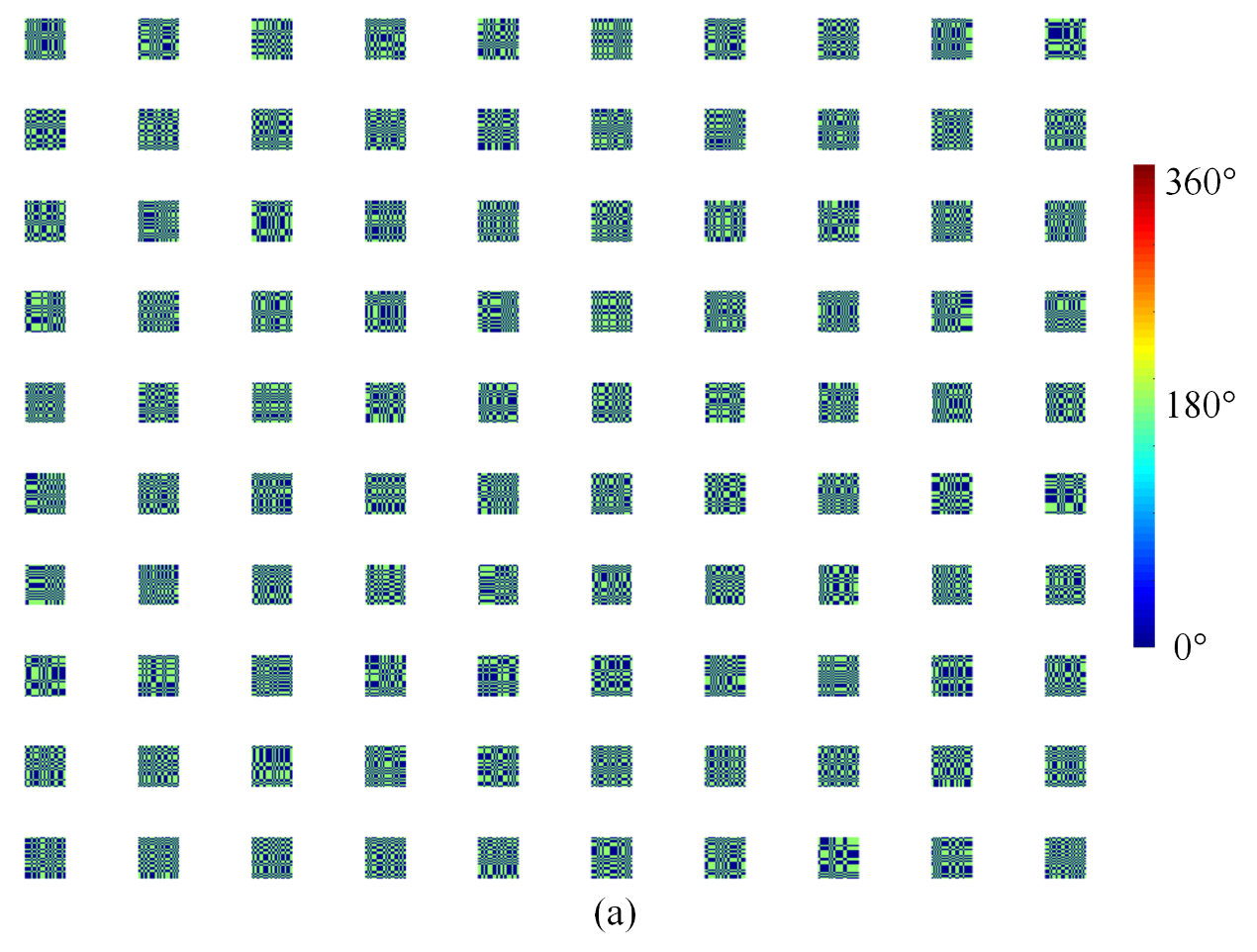

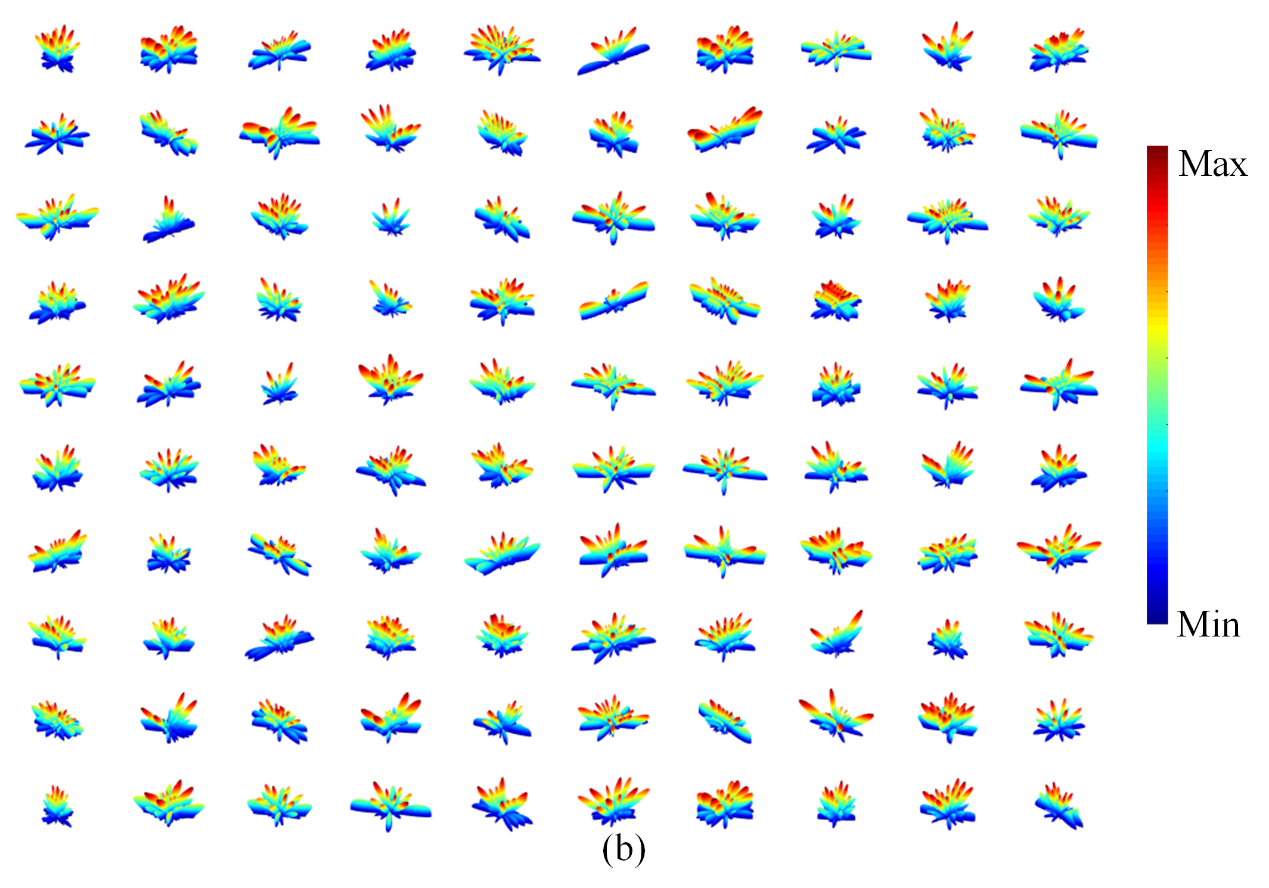

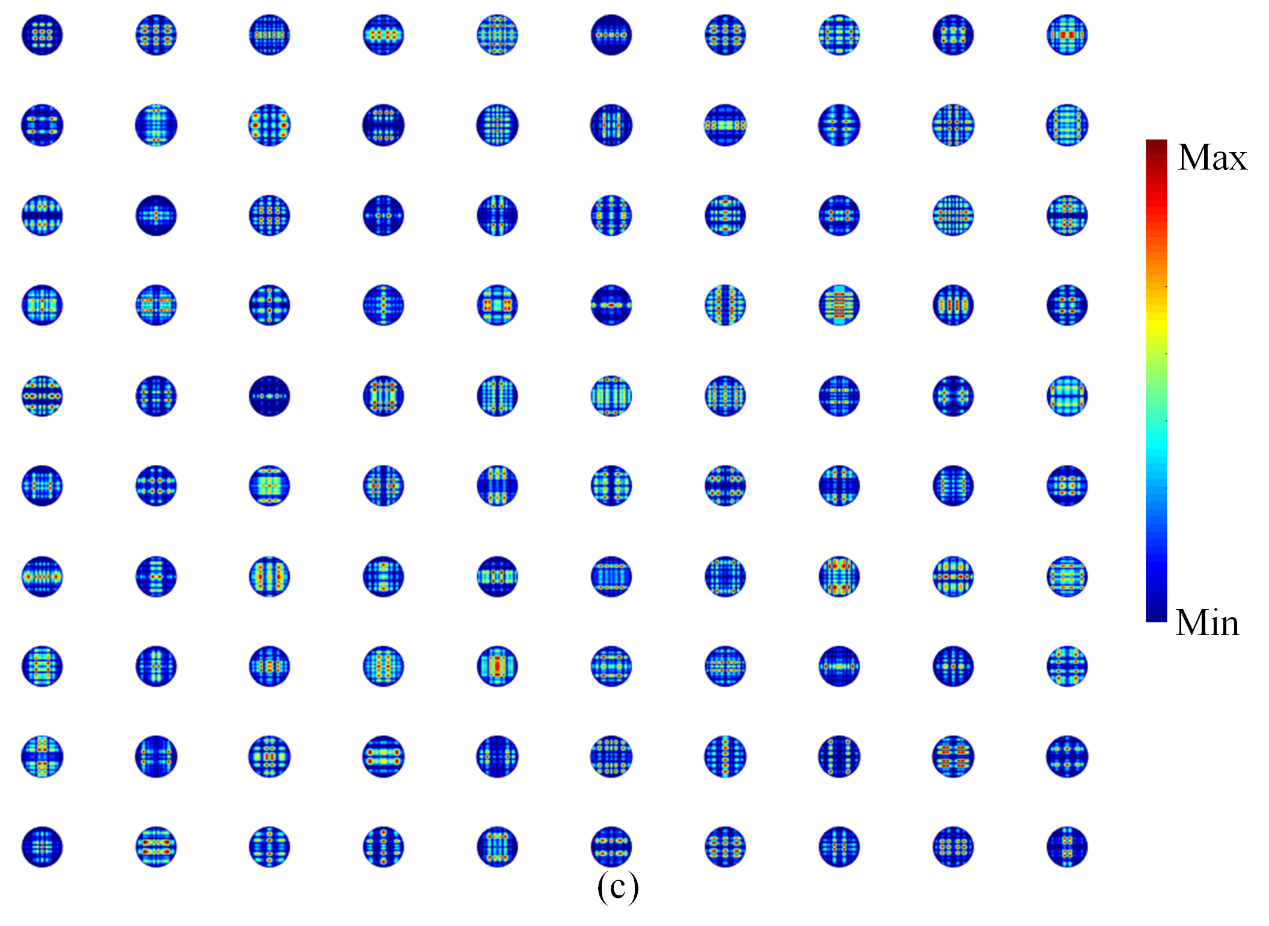


Figure S2 The XOR logic empowered multi-beam control: (a) phase profiles at different coding sequences; (b) 3D far-field scattering patterns at different phase profiles; (c) Overhead cross-section of far-field scattering at different phase profiles.

The above demonstrates random beamforming, showing that different beams can be customized through the arrangement of different coding sequences. However, XOR logic control cannot fully customize arbitrary beams, as it is constrained by certain limiting conditions. Mathematically, the necessary condition for forming an effective beam is that the target phase distribution matrix *S* must satisfy a rank of 1 (i.e., rank(*SRA*)=1) or be approximable within an acceptable error via rank approximation. This implies that the phase distribution can be decomposed into two additive terms: one dependent solely on the row index and the other solely on the column index. In previous research, scholars proposed the system parameter boundary *K* value as a quantitative criterion for this condition.

$$K=\frac{s_{11}}{\sum_{i}s_{ii}}$$

where *S_ii_* are the singular values obtained from the singular value decomposition (SVD) of the target reflection coefficient matrix *S*, and *S*_11_ is the largest singular value. The *K* value indicates how closely the target phase distribution matrix approximates its best rank-1 approximation matrix. *K* ≈ 1 indicates that the target matrix itself is close to rank one and can be perfectly realized by row-column control. *K* << 1 indicates that the target matrix is full-rank or of high order, and row-column control will introduce significant approximation errors. The *K* value is the core metric that transforms the "constraints of row-column control" from a qualitative description into a quantitative assessment: a high *K* value (>0.8) implies feasibility and good performance; a low *K* value (<0.5) suggests infeasibility or poor performance. Certainly, if more refined control is desired, the distributed control method can be combined by dividing the system into subregions for row-column cross control, thereby achieving closer approximation of specific beams.

Moreover, Programmable coding metasurfaces are generically expected to enable arbitrary wave manipulations. This issue can be further addressed through both algorithmic optimization and distributed control approaches.

Regarding the algorithmic optimization approach, XOR logic control can achieve different beams through different coding sequences. Therefore, optimization algorithms from digital control (such as random search, genetic algorithms, etc.) can be employed. By directly optimizing the row and column control vectors, the target phase distribution can be approximated. This increases the computational complexity of the control logic but does not add to the number of physical control lines. Thus, an optimization algorithm is used to optimize the encoding sequence in order to approximately generate the desired beam.

Regarding the distributed operations, a distributed row-column control method can be employed, dividing a large reflective array into multiple subregions. Each subregion independently utilizes row-column control to target different beams or achieve distinct local wavefronts. For example, to give the simplest illustration: the left half of an array forms a beam pointing in the direction (θ₁, φ₁), while the right half forms another beam pointing in (θ₂, φ₂). For each subregion, as long as the target beam satisfies the local separability condition [4] within that region, row-column control remains effective. Based on this approach, as illustrated in Figure S3, by dividing the array into *P* subregions, it is theoretically possible to independently generate *P* beams. The performance of each beam approximates that achieved when the corresponding subregion uses row-column control independently. For more complex beam requirements, such as scenarios needing specific coverage shapes (e.g., cellular sectors), the beam directions and widths of multiple partitions can be jointly optimized. The target shape can then be approximated through incoherent superposition. The advantage of this approach is that within each subregion, the number of control lines is still reduced from *Ns*×*Ms* to *Ns*+*Ms* (where *Ns* and *Ms* are the number of rows and columns in the subregion). Although the total number of lines increases compared to a single global row-column control scheme, it remains significantly lower than the *N* × *M* lines required for per-element control across the entire array.


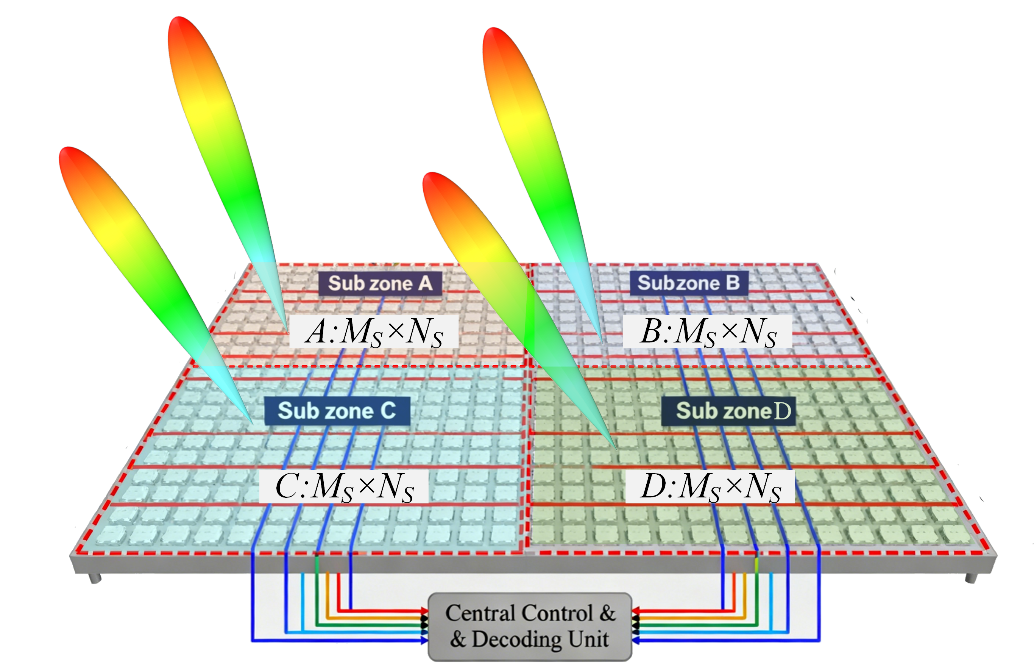


Figure S3 Distributed Control Schematic

**Supplementary Note 4.** **Metasurface empowered multi-target communication**

Here, we propose a hypothetical application scenario, that is, real-time switching multi-target communication scenario. According to Figure S2, the metasurface can control the 2D beam scattering and the far-filed beam can be customized in different scenarios. Figure S4 shows the wireless communication scenario and the number of communication targets changes in real time. Therefore, the metasurface needs to switch the scattering beam state in real time to adapt to different communication targets. The state of metasurface can be changed by control chips such as FPGA, Arduino, computer and so on. In the future, the metasurface could be further integrated with external sensors and combined with technologies such as machine learning to achieve intelligent beam steering.


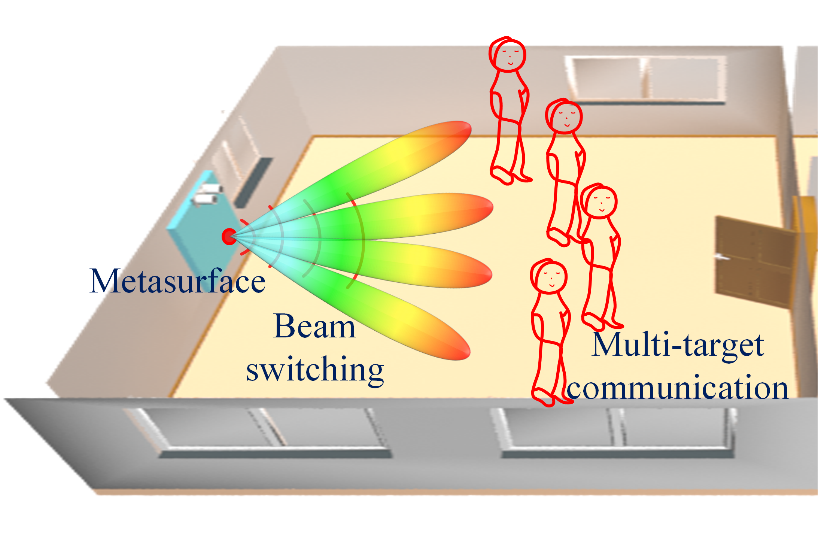


Figure S4 The multi-target communication scenario.

**Supplementary Note 5. The biasing line network of engineering drawings**

The metasurface prototype is fabricated by commercial Printed Circuit Board (PCB) technology. The engineering drawings are established by Autodesk Computer Aided Design. The top layer is shown in Figure S5(a), in which the structure pattern also is the feeding port. The bottom layer is shown in Figure S5(b), in which the reflective backplane with slits is also a feeding port.


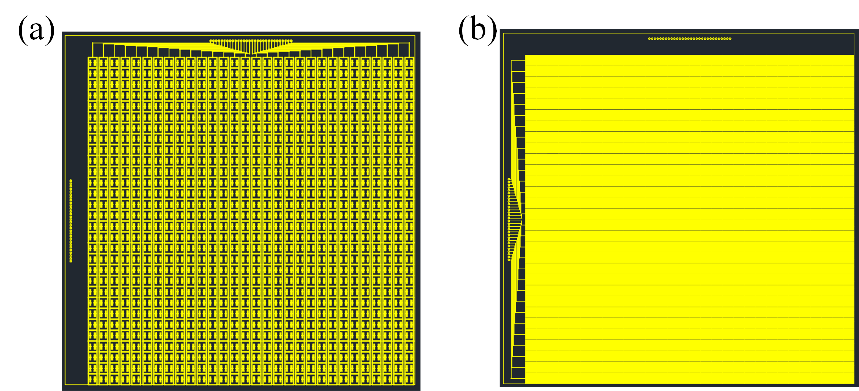


Figure S5 The engineering drawings of fabricated metasurface

**Supplementary Note 6. Analysis of power consumptions**

Here we present a comparative analysis of power consumption between conventional single-pixel control and XOR-logic (row–column-interlaced) control. First, we break down the power consumption into two parts: the power dissipated on the bias lines and the power consumed by the drive circuits. The power dissipated on the bias lines arises mainly from losses in the PIN diodes, the unit-cell structures, the substrate, and the feed network. The drive circuits typically include digital-to-analog converters (DACs) or digital control logic. Digital control logic dissipates negligible power and can be ignored. Therefore, only the DAC case is discussed here.

Taking analog control as an example, the power consumption of each DAC, *P_DAC_*, depends on its resolution and sampling rate. A typical value is 1-10 mW per channel. Here we use the average value of 5 mW per channel for estimation. The metausrface consists of 30×30 meta-atoms arrays. The traditional single-pixel scheme requires 900 DACs, giving a total drive power of 4.5 W, whereas the row-column scheme needs only 60 DACs, reducing the drive power to 0.3 W.

Since the metasurface is fabricated with copper and low-loss F4B substrate, the unit-cell dissipation is negligible and will not be discussed further. We therefore focus on calculating the power dissipated by the diodes. The PIN diode is SMP1320-079LF (from Skyworks). According to the I-V curve in the datasheet, when the diode current is 1 mA the voltage is about 760 mV. Therefore, the operating power for a single diode is 0.001 A × 0.76 V = 0.76 mW. Under single-pixel control (900 PIN diodes) the diode loss is 0.76 mW × 900 = 0.684 W, whereas under row–column control (60 PIN diodes) it drops to 0.76 mW × 60 = 0.0456 W.

Based on the above analysis, it is evident that row-column control offers substantial advantages in both device dissipation and control-port power. In the following, we integrate these various loss components and discuss them from a broader, qualitative perspective.

In traditional single-pixel control, each unit cell requires its own control line; an *M* × *N* array therefore needs *M* × *N* bias lines and the same number of drive circuits. The power consumption of traditional single-pixel control *P*_tradition_ can be expressed by Equation (S1).

$$P_{\text{tradition }}\propto M\times N\times\left(P_{\text{control}}+P_{\text{drive }}\right)$$

In which, *P*_control_ is bias lines and *P*_drive_ is drivers. In XOR-logic control, only *M* + *N* bias lines (rows and columns) are required. The corresponding power consumption *P*_XOR_ is then given by Equation (S2).

$$P_{\text{XOR }}\propto(M+N)\times\left(P_{\text{control}}+P_{\text{drive }}\right)$$

XOR-logic control reduces power loss by cutting the number of bias lines and drivers. In this work, both the row and column counts are 30 (i.e., *M* = 30, *N* = 30), so the drive-circuit power can theoretically be reduced by more than 90 %.

Furthermore, we simulated the power consumption in the circuit. We utilized ADS (Keysight Advanced Design System) to extract specific numerical values of this I-V characteristic curve and incorporated them into the PySpice library for circuit-level simulation to validate our design. The PySpice library comprehensively accounts for fluctuations within the circuit. Figure S6 simulates the power characteristics of the circuit. Figure S6(a) demonstrates the I-V characteristic curve and its voltage fluctuation range, assuming the voltage fluctuates around 760 mV. Figure S6(b) illustrates the corresponding power consumption calculation chart. Through random simulation of power consumption variations in relevant units, the simulated single-point control power consumption is approximately 0.724 W, while the simulated XOR logic control power consumption is about 0.048 W, resulting in approximately 93.3% power savings.


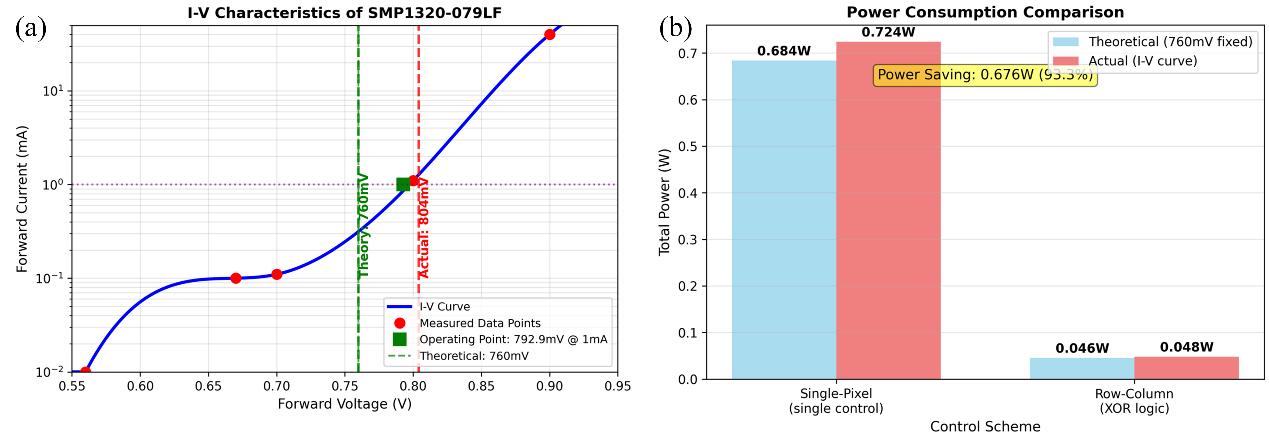


Figure S6 Power Consumption Circuit Simulation: (a) I-V Characteristics and Component Variations; (b) Power Consumption Comparison.

**Supplementary Note 7. Analysis of Frequency Offset**

The observed ~0.3 GHz discrepancy between the simulated (5.8 GHz) and measured (6.1 GHz) working frequencies in a metasurface transceiver can arise from several physical factors beyond fabrication tolerances and diode parasitics. Below is a detailed breakdown of potential contributors:

(1) Biasing Line Asymmetry

The DC biasing network (such as the traces that supply voltage to PIN diodes) can introduce unintended inductance (*L*) and capacitance (*C*) due to two main factors: trace length mismatch, which causes asymmetric routing to alter the effective impedance observed by each unit cell, and ground return path coupling, where poor grounding or unequal return paths lead to parasitic LC effects. This additional parasitic *LC* further affects the resonance of the complementary Electric-LC (cELC) elements by shifting their effective resonant frequency. For instance, a 1-mm trace asymmetry at 10 GHz can result in an approximate 0.1-0.2 GHz frequency shift because of the added inductance.

(2) Substrate Anisotropy & Dielectric Variations

Substrate anisotropy and dielectric variations impact cELC resonators. Materials like F4B or Rogers can show direction-dependent permittivity due to anisotropy from fiberglass weave or filler alignment, and commercial substrates have ±5-10% dielectric constant tolerance (e.g., F4B’s εᵣ = 2.65 ± 0.2). For a cELC resonator, frequency scales as *f*∝1/*sqrt*(*ε_eff_*), so a 5% increase in εᵣ (e.g., 2.65–2.78) lowers *f* by ~2.5% (around 0.15 GHz at 6 GHz). Also, simulations assume homogeneous εᵣ, but real substrates may have local εᵣ gradients from lamination or moisture absorption, causing measurement - simulation mismatch.

(3) Inter-Element Coupling & Mutual Impedance

Inter - element coupling and mutual impedance affect cELC elements. Closely spaced cELC elements (around *λ*/2) show mutual inductance and capacitance, changing individual resonance, and full - wave solvers might underestimate this coupling if mesh granularity is not sufficient. Moreover, in large arrays with more than 16 elements, collective modes arise, shifting the dominant resonance frequency.

(4) Diode Nonlinearity & Package Effects

Diode nonlinearity and package effects influence performance. PIN diodes have voltage - dependent junction capacitance (*C_j_*)), and a 1 - pF increase in *C_j_* (such as due to bias drift) can lower the frequency *f* by around 0.1 GHz. Additionally, package parasitics like bond wires (about 0.5 nH) and pad capacitance (about 0.2 pF) introduce unintended inductance and capacitance.

(5) Fabrication Imperfections

Fabrication imperfections affect cELC elements. Etching tolerance, with ±20 μm deviations in cELC gap width (for example, a designed 200 μm gap becoming 180 μm in fabrication), alters capacitance by around 10%. Also, layer misalignment, involving vertical offsets between metallization layers, changes the effective cELC geometry.

Furthermore, we systematically investigated the influence of several design parameters through a detailed simulation sweep. Figure S7 summarizes the findings: the dependence of frequency deviation on inductance (a) and capacitance (b), the effect of substrate permittivity (c), and the role of inter‑element coupling across different array sizes (d). Each parameter was methodically evaluated, and the results conclusively show that any such variation induces a consistent shift in the S‑parameter response.


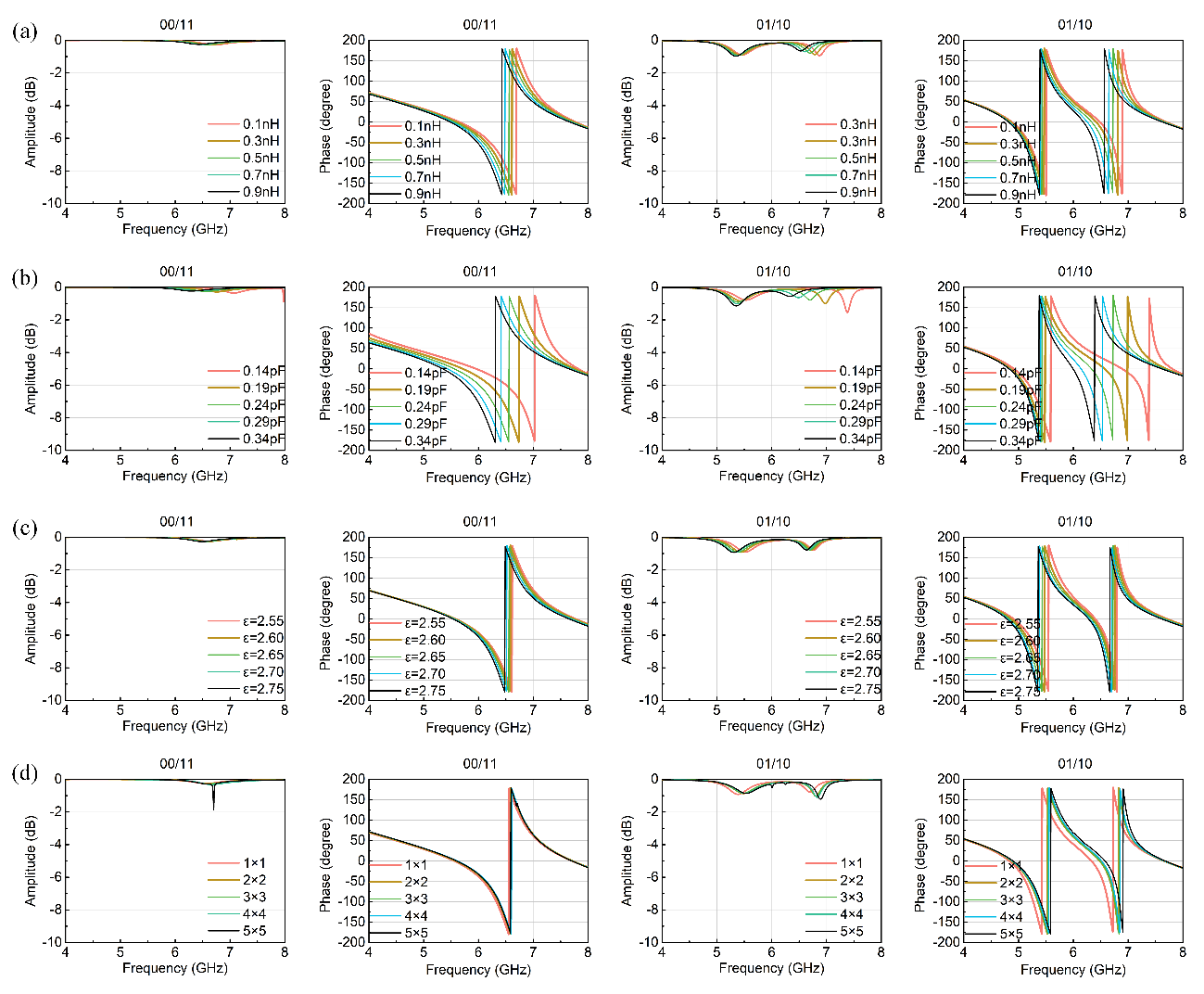


Figure S7 Influence of design parameter variations on s-parameter response: (a) Inductance variation; (b) Capacitance variation; (c) Dielectric constant variation; (d) unit cell variation.

**Supplementary References**

[1] Menzel C, Rockstuhl C, Lederer F. Advanced Jones calculus for the classification of periodic metamaterials[J]. Physical Review A, 2010, 82(5): 053811.

[2] Jones R. A new calculus for the treatment of optical systems I. description and discussion of the calculus[J]. Journal of the Optical Society of America, 1941, 31(7): 488-493.

[3] Ding X, Monticone F, Zhang K, et al. Ultrathin Pancharatnam–Berry metasurface with maximal cross‐polarization efficiency[J]. Advanced Materials, 2015, 27(7): 1195-1200.

[4] Artiga, Xavier. Row–column beam steering control of reflectarray antennas: benefits and drawbacks [J]. IEEE Antennas and Wireless Propagation Letters 17.2 (2017): 271-274.
